# Editorial: The impact of art therapy on mental health and well-being

**DOI:** 10.3389/fpsyt.2023.1275915

**Published:** 2023-09-05

**Authors:** Helena José, Joao Apostolo, Luciano Magalhães Vitorino, Luis Manuel Mota de Sousa

**Affiliations:** ^1^The Health Sciences Research Unit, Coimbra Nursing School, Coimbra, Portugal; ^2^Department of Nursing, Atlantic University Higher Institution, Atlântica University, Barcarena, Portugal; ^3^Coimbra Nursing School, Coimbra, Portugal; ^4^Portugal Centre for Evidence-Based Practice: A Joanna Briggs Institute Centre of Excellence, Coimbra, Portugal; ^5^Faculty of Medicine of Itajubá-Afya Group, Itajubá, Minas Gerais, Brazil; ^6^Comprehensive Health Research Centre, University of Evora, Evora, Portugal

**Keywords:** art therapy, approach, integrative, health, well-being

We are currently observing a concerning escalation in mental health issues. Consequently, it's crucial to tailor interventions that enhance mental health and well-being, as societies acknowledge the profound influence of psychological well-being on overall life quality.

Indeed, we are in an era where traditional therapeutic methods are being supplemented by integrative interventions. Art therapy is emerging as a promising and increasingly significant approach, especially for individuals with dementia. It's gaining substantial recognition as an effective and suitable therapeutic intervention for mental health conditions. Art therapy operates on the premise that creative expression can foster emotional well-being. “*The Impact of Art Therapy on Mental Health and Well-being*” is a compilation of papers discussing integrative care, primarily employing art as an innovative approach. It utilizes quantitative and/or qualitative methodologies to explore art therapy and the artistic heritage available for promoting mental health and well-being.

The primary objective of this topic was to provide a comprehensive view of art's role in promoting mental health and well-being across various settings. The articles published under this topic elucidate the specific mechanisms through which art therapy facilitates emotional expression, self-discovery, and resilience development among diverse individuals, primarily in educational or care settings.

We anticipate that the findings of this topic will significantly contribute to the broader context of care in Psychiatry, particularly in mental health and the field of integrative therapies, aligning with the World Health Organization's appeal.

The articles published on this topic unequivocally suggest that art therapy can bridge the gap between conventional therapeutic modalities and creative expression. By establishing a clear understanding of art's therapeutic benefits, we can foster collaboration between mental health professionals and artists, promoting an interdisciplinary approach to treatment and support across various contexts.

This topic has garnered 16k views and 2,339 downloads, indicating immense interest. It involved 52 authors, with 18 articles submitted and 14 published. These articles underscore the role of creativity and art in promoting well-being and mental health in education and healthcare. By clarifying the psychological processes involved in art therapy, this topic aims to destigmatize mental health issues and advocate for art as a valuable tool for self-discovery and emotional growth.

Moreover, the articles published here can inform policy decisions and facilitate the integration of art therapy into mainstream mental health care. They can also enlighten policymakers to recognize art therapy as a valid and beneficial therapeutic modality, potentially broadening its accessibility to a wider range of individuals in need.

In conclusion, as editors of the topic “*The Impact of Art Therapy on Mental Health and Well-Being*,” we hope to provide a comprehensive overview of art therapy's impact on mental health and well-being. By contextualizing the findings, we aspire to advance mental health care and promote holistic healing through the transformative power of art. With increased awareness and appreciation of art therapy's potential, we can progress toward a society that prioritizes mental health and adopts strategies to assist individuals in any situation or context.

## Author contributions

HJ: Writing—original draft. JA: Supervision, Writing—review and editing. LV: Writing—review and editing. LS: Writing—review and editing.

